# Questionnaires on stigmatizing attitudes among healthcare students in Taiwan: development and validation

**DOI:** 10.1186/s12909-020-1976-1

**Published:** 2020-02-27

**Authors:** Hui-Ing Ma, Chu-En Hsieh

**Affiliations:** 1 Department of Occupational Therapy, National Cheng Kung University, 1 University Road, Tainan, 701 Taiwan; 2 National Cheng Kung University, Institute of Allied Health Sciences, 1 University Road, Tainan, 701 Taiwan

**Keywords:** Anti-stigma, Attitudes, Psychometric testing, Stigma, Health professionals

## Abstract

**Background:**

People may be stigmatized if they have mental illness, emotional and behavioral disorders (EBD), or physical or intellectual disabilities. Being stigmatized adversely affects one’s psychological well-being and quality of life. While occupational therapists frequently work with people with EBD and disabilities, all healthcare practitioners may encounter these populations, and stigmatizing attitudes of healthcare professionals towards such clients can negatively affect the therapeutic relationship, evaluation, and treatment. Therefore, understanding attitudes of healthcare students—as future practitioners in all fields of healthcare—towards people in this regard is fundamental to the future implementation of anti-stigma programs. We aimed to develop and test questionnaires for examining stigmatizing attitudes of healthcare students towards people with mental illness or disabilities and children with EBD.

**Methods:**

A literature review was conducted to identify surveys related to attitudes towards people with mental illness, EBD, and disabilities. Items that were pertinent to the concept of stigma were selected and modified to fit into the Taiwanese context. A total of 336 students from departments of occupational therapy, physical therapy, nursing, and medicine in 7 universities across Taiwan completed the questionnaires. Item analysis and factor analysis were used to examine the reliability and validity of the questionnaires. Gender differences were also considered.

**Results:**

Factor analyses of the three questionnaires yielded factor structures that explained 61.34 to 67.15% of the variance, with Cronbach’s α values ranging from 0.71 to 0.89. The Questionnaire on Stigmatizing Attitudes Towards Mental Illness consisted of 16 items with 4 subscales: deviant behavior, social isolation, negative stereotype, and self-stigma. The Questionnaire on Stigmatizing Attitudes Towards Children with EBD consisted of 14 items with 3 subscales: rejective attitude, negative stereotype, and deviant behavior. The Questionnaire on Stigmatizing Attitudes Towards Disabilities consisted of 10 items with 3 subscales: positive stereotype, negative stereotype, and pessimistic expectation. In addition, men had slightly higher stigmatizing attitudes than women.

**Conclusions:**

The results showed satisfactory factor structures and internal consistency, and thus support the use of these questionnaires to understand attitudes of healthcare students towards these populations. In addition, particular attention should be paid to gender differences in stigmatizing attitudes of healthcare students.

## Background

Stigma refers to negative attitudes and discriminatory behaviors towards people with devalued characteristics that result, in part, from a lack of knowledge about those characteristics [1]. Among the possible stigmatized attributes, mental illness, emotional and behavioral disorders (EBD), and disabilities are conditions that healthcare professionals are likely to encounter during clinical practice. Attitudes of healthcare professionals towards people living with mental illness are important to building therapeutic rapport, as well as to the evaluation and intervention processes. However, research has revealed mixed attitudes of healthcare professionals towards these populations [2–5] and some patients with mental illness even reported stigma-related experience when interacting with healthcare professionals [6–8]. Such experiences of stigma are likely to aggravate patients’ feelings of rejection and incompetence, and thus are detrimental to patients’ treatment-seeking and ongoing participation in treatment [9]. Therefore, examining the stigmatizing attitudes of healthcare students towards these populations is a crucial step in planning educational interventions to enhance stigma awareness and reduce stigmatizing attitudes and behaviors (i.e., anti-stigma programs) for these future professionals.

Mental illness has long been stigmatized [10]. Common stereotypes about people with mental illness are that they are dangerous, unpredictable, and incompetent [11]. Such negative stereotypes are highly associated with fear and may result in discriminatory behaviors towards people with mental illness such as avoidance and withdrawal. For example, members of the general public do not want to have mental health institutes in their neighborhood; employers refuse to hire individuals with mental illness. If people with mental illness agree with the stereotypes and apply the labels to themselves (i.e., self-stigma), the consequent diminished self-esteem and self-efficacy would further restrict their efforts to seek jobs, treatment, and recovery.

In addition to adults with mental illness, children with EBD (e.g., autism, attention deficit hyperactivity disorder [ADHD]) may also be stigmatized [12]. Common stereotypes include that they are troublemakers and less academically and socially able than their peers. These children may be despised and rejected by peers at school. Parents of children with EBD are also likely to experience stigma by association [13]. That is, the parents are blamed for their children’s problems. The stigma related to children with EBD may deter their parents from seeking diagnosis and professional help. Moreover, as childhood is a key period for the development of self and the capacity to have close emotional and social bonds with others, being stigmatized during childhood may have a lasting negative impact on a child’s lifelong development [14].

In addition to mental illness, people with physical and intellectual disabilities are also targets of stigmatization. Seeing people with physical disabilities (e.g., amputee, stroke, cerebral palsy, spinal cord injury) may trigger a threat to body image and existential anxiety, thus eliciting uncomfortable feelings in able-bodied individuals and the desire to withdraw from such encounters [15]. Similarly, with regard to people with intellectual disabilities, although they may be viewed as innocent, they are also perceived to be incapable, dependent, and lacking the potential to change [16]. These findings indicate that people with physical or intellectual disabilities are perceived as a burden to their families and society. Their opportunities to fully integrate into the community life are constrained.

People with mental illness or disabilities and children with EBD are usually in need of healthcare and rehabilitation services to assist them in adapting to their difficulties and achieving their full potential. Attitudes of healthcare professionals towards these people and their families in this process thus play a critical role in their motivation and intention to become involved in therapy. Negative, stigmatizing attitudes of professionals are barriers to the building of therapeutic relationships and the delivery of quality services [12].

Students entering healthcare professions are also members of the general public who may share the public stigma rooted in our sociocultural system [14]. While all healthcare practitioners are likely to interact with members of these often-stigmatized populations, the practice of occupational therapy is primarily concerned with people with mental illness, children with EBD, and people with physical or intellectual disabilities. Therefore, it is important to understand the stigmatizing attitudes of healthcare students, including students of occupational therapy, towards people with mental illness, EBD, and disabilities. The purpose of this study was to develop and validate questionnaires to evaluate the stigmatizing attitudes of healthcare students towards these populations.

## Methods

This paper reports on the tasks completed in the 1st year of a three-year prospective project aiming to develop an anti-stigma program for occupational therapy students. We developed questionnaires to evaluate stigmatizing attitudes towards the populations that occupational therapists commonly treat in practice. The questionnaires, however, were administered to not only occupational therapy students but also to other healthcare students, with the goal to obtain a broad baseline understanding and to serve as a reference for evaluation of the anti-stigma program to be developed in the future.

### Item development and selection

A literature review was conducted to identify existing questionnaires pertaining to the measurement of attitudes towards people with mental illness, children with EBD, and people with disabilities. Items of the relevant questionnaires were reviewed, and those pertaining to stereotypes, prejudice, and discrimination were included and modified to fit into the Taiwanese context. For mental illness, we included 30 items that were adapted from the Community Attitudes Toward the Mentally Ill (CAMI) [17] and the Community Attitude Survey to Mental Illness [18]. For children with EBD, we included 20 items that were adapted from the Attitudes About Child Mental Health Questionnaire (ACMHQ) [19] and the Peer Mental Health Stigmatization Scale (PMHSS) [20]. For people with disabilities, we included 16 items adapted from the Attitudes to Disability Scale (ADS) [21]. A 6-point Likert scale was used for all the questionnaires, with 1 indicating “strongly disagree” and 6 “strongly agree.” Higher scores represent more negative stigmatizing attitudes (items phrased in the opposite direction were reverse coded). In this study, we presented the average score of the items on each questionnaire (possible range of 1 to 6).

### Participants

Our sample size was determined based on two perspectives: (1) the appropriate minimum size for the conditions required for factor analysis, and (2) the minimum necessary to be representative of the population of interest. Regarding the first, according to Fabrigar & Wegener [22], under moderately good conditions (communalities of .40 to .70 with at least 3 measured variables loading on each factor), a sample of at least 200 is adequate. Regarding the second, we estimated our sample size based on the data from the Ministry of Education in Taiwan (https://udb.moe.edu.tw/DataDownload) indicating that a total of 36,019 medical and allied health college students were enrolled in 2014 (the most updated data at that time). Sample size estimation with a margin of error of 5% and a confidence level of 95% of the population indicated that 381 respondents were required.

We recruited participants from 7 universities that have departments of medicine, nursing, occupational therapy, and physical therapy, aiming to have 5 to 10 students in each of the years from Year 1 to Year 4. Until June 30, 2017, questionnaires were mailed to 413 students and responses were obtained from 336 students (response rate: 81.36%). Table 1 presents the student numbers by gender, year, and department.

**Table 1:** Social demographics and academy data of study participants

	Women (*n*, %)	Men (*n*, %)	Total (*n*, %)
	239 (71.1%)	97 (28.9%)	336 (100%)
Department			
OT	156(46.4%)	41(12.2%)	197(58.6%)
PT	37(11.0%)	35(10.4%)	72(21.4%)
Nursing	23(6.8%)	5(1.5%)	28(8.3%)
Medicine	23(6.8%)	16(4.8%)	39(11.6%)
Year of study			
1st	52(21.8%)	20(6.0%)	72(21.4%)
2nd	66(19.6%)	34(10.1%)	100(29.8%)
3rd	70(20.8%)	21(6.3%)	91(27.1%)
4th	51(15.2%)	22(6.5%)	73(21.7%)

*OT* Occupational Therapy, *PT* Physical Therapy

### Data analysis

SPSS version 17 was used for item analysis, factor analysis, and t-test. An item was deleted if (1) absolute values of skewness were close to 1 or − 1, (2) comparisons of extreme groups by t-tests led to *p*-values greater than 0.001, (3) corrected item-total correlation values were less than 0.3, and (4) factor loading values were less than 0.3. Exploratory factor analyses (principal components) were then conducted on the remaining items to derive the subscales of each questionnaire. Cronbach’s α was used to estimate the internal consistency of the questionnaires. In addition, given the existing evidence about gender differences in attitudes [23, 24], we used t-tests to compare attitudes between women and men. We calculated the average score per item on each subscale and then averaged again by subscale to obtain the final score for each questionnaire.

## Results

### Instrument structure and internal consistency

According to our aforementioned criteria, 18, 14, and 10 items were retained for the questionnaires on stigmatizing attitudes towards mental illness, EBD, and disabilities, respectively. The Kaiser-Meyer-Olin (KMO) measure of sampling adequacy was 0.885, 0.900, and 0.722, respectively, indicating the appropriateness of the factor analysis for this data set. In addition, Bartlett’s tests of sphericity were significant (χ^2^ [120] = 2728.554, χ^2^ [45] = 998.261, χ^2^ [91] = 2105.852, respectively), supporting the factorability of the correlation matrix.

For the Questionnaire on Stigmatizing Attitudes Towards Mental Illness, the factor analysis of the 18 items, using varimax rotation to account for the relationship among the factors, yielded a five-factor structure that explained 69.18% of the variance of the data. However, factor 5 consisted of only two items, and thus we deleted these two items and ran the factor analysis again. The factor analysis of the remaining 16 items yielded a four-factor structure that explained 67.15% of the variance of the data (Table 2). Factor 1 (deviant behavior, 5 items) explained 23.33% of the total variance; factor 2 (social isolation, 3 items) explained 17.54%; factor 3 (negative stereotype, 5 items) explained 14.12%; and factor 4 (self-stigma, 3 items) explained 12.16%. Cronbach’s α was 0.89 for the entire questionnaire.

**Table 2:** Factor loadings, communalities, and Cronbach’s α for questionnaire on stigmatizing attitudes towards mental illness

	Deviant Behavior	Social Isolation	Negative Stereotype	Self-stigma	Communality
21. I think persons with mental illness would talk gibberish.	.889				.848
18. I think people with mental illness would mumble to themselves.	.884				.829
20. I think people with mental illness would indulge in flights of fancy.	.844				.738
19. I think people with mental illness would shout and scream.	.810				.780
17. I think people with mental illness are dangerous.	.555				.678
09. People with mental illness living in the community would endanger local residents.		.808			.734
08. It is frightening to have people with mental illness living in residential neighborhoods.		.767			.739
10. Mental health facilities should be kept out of residential neighborhoods.		.760			.664
16. I think people with mental illness have dementia.			.654		.664
14. Anyone with mental illness should be excluded from political campaigns.			.653		.591
01. People with mental illness are less capable than others.			.639		.546
15. I think people with mental illness usually appear unkempt.			.597		.581
12. I do not believe anything people with mental illness say.			.505		.515
04. I would feel ashamed if I visit psychosomatic clinics.				.790	.667
06. If I have mental illness, this means I am not “normal.”				.716	.596
07. I won’t let people know if there is a person with mental illness in my family.				.685	.574
Rotation sums of squared loadings’ total	3.733	2.806	2.259	1.945	
Cronbach’s α	.909	.815	.763	.676	
	.891				

Regarding the Questionnaire on Stigmatizing Attitudes Towards Children with EBD, the factor analysis of the 14 items yielded a three-factor structure that explained 62.64% of the variance of the data (Table 3). Factor 1 (rejective attitude, 4 items) explained 25.08% of the total variance; factor 2 (negative stereotype, 7 items) explained 23.57%; and factor 3 (deviant behavior, 3 items) explained 13.99%. Cronbach’s α was 0.86 for the entire questionnaire.

**Table 3:** Factor loadings, communalities, and Cronbach’s α for questionnaire on stigmatizing attitudes towards children with EBD

	Rejective Attitude	Negative Stereotype	Deviant Behavior	Communality
09. I would rather that relatives who have children with EBD do not attend family gatherings.	.837			.710
08. It would be difficult for me to accept having a relative whose child has EBD.	.823			.708
10. I would think less positively of a child with EBD.	.692			.628
11. I would rather not work with a teenager with EBD.	.589			.636
19. I think that children with EBD are not as good as other children at taking care of themselves.		.779		.617
18. I think that children with EBD do not behave as well as other children.		.758		.689
12. If I were a boss, I would rather not hire a teenager with EBD.		.600		.585
17. It is a bad idea to give a part-time job to a teenager with EBD.		.587		.652
15. I think that children with EBD are not as trustworthy as other children.		.554		.592
14. I think that children with EBD are dangerous.		.530		.543
20. I would be afraid of someone if I knew that they had EBD.		.473		.484
01. Children with EBD would hurt themselves or other children.			.775	.679
03. Children with EBD are troublemakers.			.713	.699
02. When children have problems with their emotions and behavior, it is because their parents did not raise them properly.			.604	.546
Rotation sums of squared loadings’ total	3.511	3.299	1.959	
Cronbach’s α	.840	.854	.613	
	.895			

*EBD* Emotional and behavioral disorders

Regarding the Questionnaire on Stigmatizing Attitudes Towards Disabilities, the factor analysis of the 10 items yielded a three-factor structure that explained 61.34% of the variance of the data (Table 4). Factor 1 (positive stereotype, 4 items) explained 29.17% of the total variance; factor 2 (negative stereotype, 3 items) explained 17.47%; and factor 3 (pessimistic expectation, 3 items) explained 14.70%. Cronbach’s α was 0.71 for the entire questionnaire.

**Table 4:** Factor loadings, communalities, and Cronbach’s α for questionnaire on stigmatizing attitudes towards disabilities

	Positive Stereotype	Negative Stereotype	Pessimistic Expectation	Communality
08. Having a disability can make someone a wiser person.	.845			.729
09. Some people achieve more because of their disability.	.843			.712
07. Having a disability can make someone a stronger person.	.831			.704
10. People with a disability are more determined than others to reach their goals.	.802			.644
05. People with a disability are a burden on society.		.859		.755
06. People with a disability are a burden on their family.		.857		.744
01. People with a disability find it harder than others to make new friends.		.435		.323
16. People with a disability have less to look forward to than others.			.762	.601
11. People tend to become impatient with those with a disability.			.697	.521
14. People should not expect too much from those with a disability.			.499	.401
Rotation sums of squared loadings’ total	2.917	1.747	1.470	
Cronbach’s α	.861	.633	.440	
	.706			

### Gender difference

Table 5 presents the average score for each questionnaire by gender and department. Significant gender differences were found in the results of all three questionnaires, with men having higher scores than women (Mental illness: *t* = 2.01, *p* = .046; EBD: *t* = 4.34, *p* < .001; Disabilities: *t* = 2.56, *p* = .011).

**Table 5:** Mean scores for the stigmatizing attitudes questionnaires

Department	Questionnaires on stigmatizing attitudes		
	People with mental illness	Children with EBD	People with disabilities
Occupational therapy			
Women (*n* = 156)	2.47 ± 0.57	2.72 ± 0.64	3.01 ± 0.54
Men (*n* = 41)	2.57 ± 0.66	2.96 ± 0.69	3.17 ± 0.52
Physical therapy			
Women (*n* = 37)	2.38 ± 0.51	2.58 ± 0.59	2.91 ± 0.52
Men (*n* = 35)	2.78 ± 0.68	3.16 ± 0.71	3.25 ± 0.55
Nursing			
Women (*n* = 23)	2.71 ± 0.78	2.96 ± 0.74	3.06 ± 0.40
Men (*n* = 5)	2.59 ± 1.16	3.21 ± 0.94	2.98 ± 0.79
Medicine			
Women (*n* = 23)	2.39 ± 0.60	2.71 ± 0.68	3.15 ± 0.35
Men (*n* = 16)	2.46 ± 0.63	3.08 ± 0.64	3.06 ± 0.58
Total			
Women (*n* = 239)	2.48 ± 0.59	2.72 ± 0.65	3.01 ± 0.51
Men (*n* = 97)	2.63 ± 0.69	3.06 ± 0.69	3.17 ± 0.55

*EBD* Emotional and behavioral disorders

## Discussion

This paper described the development and psychometric testing of questionnaires designed to examine stigmatizing attitudes towards people with mental illness, children with EBD, and people with physical or intellectual disabilities (Additional file 1). Although the Cronbach’s α of some subscales was lower than the recommended criterion of 0.7, given that the questionnaires are at an early stage of research [25] and the number of items in the subscale is small (3 items only), we consider that the Cronbach’s α values for the overall questionnaires of 0.89, 0.90, and 0.71, respectively, suggests adequate internal consistency.

We developed the three questionnaires simultaneously because occupational therapists mainly work with these populations in practice. In comparison to some general attitude surveys (e.g., Attitude Toward Disabled Persons (ATDP) [26], Interactions with Disabled Persons Scale (IDP) [27]), our questionnaires focus on the stigma aspect, including stereotype, prejudice, and discrimination. Stigma, developed since childhood, has been reported to have a profound influence on one’s attitude and behavior [14]. Therefore, examining stigmatizing attitudes in healthcare students is a fundamental step for developing stigma awareness and future anti-stigma programs.

Each of our questionnaires addresses stigma specific to a target population and thus is more sensitive to that condition. For example, the self-stigma subscale reflects the common situation of people with mental illness internalizing the negative stereotypes and prejudice about their illness. Items in the Questionnaire on Stigmatizing Attitudes Towards Children with EBD reflect the stigma by association about families of the children. Moreover, in comparison to the ample research on healthcare professionals’ attitudes towards adults with mental illness and disabilities, only a few studies have examined professionals’ attitudes towards children with EBD. Given the rising rate of children with EBD and their under-utilization of mental health services, it is important to recognize stigma as a key impeding factor in early identification and intervention, especially for healthcare professionals [28].

Positive stereotype is one of the subscales in our Questionnaire on Stigmatizing Attitudes Towards Disabilities. Although items in this subscale, such as “Having a disability can make someone a wiser/stronger person” may sound favorable, such a description may also lead to feelings of being depersonalized [29]. That is, the person with disabilities is seen as reduced to merely their group membership rather than being seen as an individual. The subjective favorability of positive stereotype also tacitly implies some corresponding deficiency. Furthermore, positive stereotypes may be used strategically by higher-status groups to flatter subordinate group members into accepting their lower status. Therefore, we should be cautious about such statements and encourage people to perceive and acknowledge variability within people with disabilities.

In this study, the average score of items in the questionnaires ranged from about 2 to 3, suggesting that students “disagree moderately” to “disagree a little” on the negative statements. While such scores may reflect that students had low stigmatizing attitudes towards these populations, social desirability bias should be taken into account [30]. Despite this, we found gender differences, with men having higher scores than women in all the three questionnaires. The results are in line with previous findings that women had more positive attitudes than men towards people with mental illness [24] and towards people with physical disabilities [4]. The lower stigmatizing attitudes in women may correspond with a generally higher rate of social empathy, given that as the more empathic a person is, the less likely he/she holds stigmatizing attitudes towards a group [24]. In addition, the more stigmatizing attitudes in men may be attributable to traditional masculine ideals that value strength, competence, and independence [23]. The results suggest that special attention may be paid to gender differences related to empathy and values of strength, competence, and independence in the anti-stigma program to be developed in the future.

Other surveys have been developed to assess stigmatizing attitudes in healthcare professionals towards people with mental illness, such as the Mental Illness Clinicians’ Attitudes (MICA) Scale [31] and the Opening Minds Stigma Scale for Health Care Providers (OMS-HC) [32]. However, we did not use these scales because we determined that students are in a different stage involving different experiences compared to working professionals, thus some items that are related to professional practice and interaction with colleagues may not be appropriate to the student participants. In comparison with the MICA Scale and OMS-HC, our questionnaires are more general and can be administered to general populations. On the other hand, ours are not specific to healthcare providers and thus may not be sensitive to healthcare circumstances. Future research aiming to evaluate the outcome of an anti-stigma program should choose the assessment tool according to the recipients of the program.

Stigma research in Taiwan has mainly focused on self-stigma in people with mental illness [33–35]. To the best of our knowledge, only a few studies have examined the attitudes of healthcare students towards people with mental illness and physical disabilities [36, 37]. Wang et al. examined the explicit and implicit stigma toward people with mental illness in medical and non-medical students [37]. They found that the two groups had similar levels of explicit and implicit stigma at baseline. For medical students, explicit stigma significantly decreased but implicit stigma remained similar after a one-month psychiatric clerkship, while non-medical students’ levels of stigma were unchanged after 1 month. Another study of occupational therapy students’ attitudes towards individual with disabilities compared the results from students from Australia, Taiwan, the United Kingdom, and the United States [36]. The results indicated that occupational therapy students from Taiwan exhibited a higher degree of discomfort in social situations with individuals with disabilities compared to students from the other three countries. The results of these studies as well as ours confirm the existence of stigmatizing attitudes and highlight the importance of examining and addressing such attitudes, including explicit and implicit stigma, in healthcare students.

By assessing stigmatizing attitudes, this study also highlights the importance of addressing stigma-related issues in healthcare education. Many anti-stigma educational interventions have been developed and examined to reduce healthcare students’ stigmatization of people with mental illness [38–42]. However, it is also necessary to attend to stigma issues related to other clinical populations with various diseases and disabilities. Addressing stigma issues is important during students’ studies and, in particular, during clinical placement, to prepare students for positive attitudes in developing therapeutic rapport with their clients [43].

Some limitations of this study should be noted. First, although we aimed to have a representative sample of medical and allied health college students in Taiwan, students from departments of medicine and nursing were relatively few. In addition, although some healthcare professions are considered female-dominated, the number of men participating in this study was still lower than we expected. Therefore, caution should be used when generalizing the results to male students of a specific healthcare profession. Second, in this paper, we reported instrument structure and internal consistency. It should be noted that the development of a questionnaire requires continual effort. Other measurement properties have to be established too, such as test-retest reliability, criterion validity, and responsiveness [44]. In the present study, because we tested three questionnaires at a time, in order not to overburden our participants, we did not include other measures to test criterion validity. For future research, some short surveys could be included to examine construct validity. For example, the Reported and Intended Behavior Scale (RIBS) [45] that tests behavioral discrimination against people with mental health problems could be used to examine the convergent validity of our Questionnaire on Stigmatizing Attitudes Toward Mental Illness. Future research is necessary to examine psychometric characteristics for each questionnaire in more depth.

## Conclusions

People with mental illness, children with EBD, and people with disabilities often require healthcare and rehabilitation services to adapt to their difficulties and optimize their strengths. In this on-going process, attitudes of healthcare professionals are a critical factor to facilitate or impede the development of the therapeutic alliance. Therefore, understanding attitudes of healthcare students is essential for stigma awareness and reduction. This study developed and tested three questionnaires to examine stigmatizing attitudes towards these populations. The results showed satisfactory factor structures and internal consistency, and thus support the use of these questionnaires to examine healthcare students’ attitudes. In addition, the results of higher stigmatizing attitudes in men than women suggest the importance of addressing gender differences in future anti-stigma programs.

## Supplementary information

**Additional file 1.** Final version of questionnaires on stigmatizing attitudes towards mental illness, disabilities, and children with emotional and behavioral disorders

## Data Availability

The datasets used and/or analyzed during the current study are available from the corresponding author on reasonable request.

## Abbreviations

EBD: Emotional and behavioral disorders
OT: Occupational therapy
PT: Physical therapy
