# Supplementary material for: Questionnaires on stigmatizing attitudes among healthcare students in Taiwan: development and validation
Source: BMC Med Educ. 2020 Feb 27;20:59. doi: 10.1186/s12909-020-1976-1 (PMC7045580; doi:10.1186/s12909-020-1976-1)
Supplement: Supplementary file 1 — Additional file 1. Final version of questionnaires on stigmatizing attitudes towards mental illness, disabilities, and children with emotional and behavioral disorders [file 12909_2020_1976_MOESM1_ESM.docx]

Questionnaire on Stigmatizing Attitudes Towards Mental Illness

|  | *☹*  *☺* | | | | | |
| --- | --- | --- | --- | --- | --- | --- |
|  | Strongly disagree | Disagree | Slightly disagree | Slightly agree | Agree | Strongly agree |
| 01. I think people with mental illness are dangerous. | □ | □ | □ | □ | □ | □ |
| 02. I think people with mental illness would mumble to themselves. | □ | □ | □ | □ | □ | □ |
| 03. I think people with mental illness would shout and scream. | □ | □ | □ | □ | □ | □ |
| 04. I think people with mental illness would indulge in flights of fancy. | □ | □ | □ | □ | □ | □ |
| 05. I think persons with mental illness would talk gibberish. | □ | □ | □ | □ | □ | □ |
| 06. It is frightening to have people with mental illness living in residential neighborhoods. | □ | □ | □ | □ | □ | □ |
| 07. People with mental illness living in the community would endanger local residents. | □ | □ | □ | □ | □ | □ |
| 08. Mental health facilities should be kept out of residential neighborhoods. | □ | □ | □ | □ | □ | □ |
| 09. People with mental illness are less capable than others. | □ | □ | □ | □ | □ | □ |
| 10. I do not believe anything people with mental illness say. | □ | □ | □ | □ | □ | □ |
| 11. Anyone with mental illness should be excluded from political campaigns. | □ | □ | □ | □ | □ | □ |
| 12. I think people with mental illness usually appear unkempt. | □ | □ | □ | □ | □ | □ |
| 13. I think people with mental illness have dementia. | □ | □ | □ | □ | □ | □ |
| 14. I would feel ashamed if I visit psychosomatic clinics. | □ | □ | □ | □ | □ | □ |
| 15. If I have mental illness, this means I am not “normal.” | □ | □ | □ | □ | □ | □ |
| 16. I won’t let people know if there is a person with mental illness in my family. | □ | □ | □ | □ | □ | □ |

|  | Strongly disagree | Disagree | Slightly disagree | Slightly agree | Agree | Strongly agree |
| --- | --- | --- | --- | --- | --- | --- |
| 01. Having a disability can make someone a stronger person. | □ | □ | □ | □ | □ | □ |
| 02. Having a disability can make someone a wiser person. | □ | □ | □ | □ | □ | □ |
| 03. Some people achieve more because of their disability. | □ | □ | □ | □ | □ | □ |
| 04. People with a disability are more determined than others to reach their goals. | □ | □ | □ | □ | □ | □ |
| 05. People with a disability find it harder than others to make new friends. | □ | □ | □ | □ | □ | □ |
| 06. People with a disability are a burden on society. | □ | □ | □ | □ | □ | □ |
| 07. People with a disability are a burden on their family. | □ | □ | □ | □ | □ | □ |
| 08. People tend to become impatient with those with a disability. | □ | □ | □ | □ | □ | □ |
| 09. People should not expect too much from those with a disability. | □ | □ | □ | □ | □ | □ |
| 10. People with a disability have less to look forward to than others. | □ | □ | □ | □ | □ | □ |

Questionnaire on Stigmatizing Attitudes Towards Disabilities

Questionnaire on Stigmatizing Attitudes Towards Children with Emotional and Behavioral Disorders (EBD)

|  | Strongly disagree | Disagree | Slightly disagree | Slightly agree | Agree | Strongly agree |
| --- | --- | --- | --- | --- | --- | --- |
| 01. It would be difficult for me to accept having a relative whose child has EBD. | □ | □ | □ | □ | □ | □ |
| 02. I would rather that relatives who have children with EBD do not attend family gatherings. | □ | □ | □ | □ | □ | □ |
| 03. I would think less positively of a child with EBD. | □ | □ | □ | □ | □ | □ |
| 04. I would rather not work with a teenager with EBD. | □ | □ | □ | □ | □ | □ |
| 05. If I were a boss, I would rather not hire a teenager with EBD. | □ | □ | □ | □ | □ | □ |
| 06. I think that children with EBD are dangerous. | □ | □ | □ | □ | □ | □ |
| 07. I think that children with EBD are not as trustworthy as other children. | □ | □ | □ | □ | □ | □ |
| 08. It is a bad idea to give a part-time job to a teenager with EBD. | □ | □ | □ | □ | □ | □ |
| 09. I think that children with EBD do not behave as well as other children. | □ | □ | □ | □ | □ | □ |
| 10. I think that children with EBD are not as good as other children at taking care of themselves. | □ | □ | □ | □ | □ | □ |
| 11. I would be afraid of someone if I knew that they had EBD. | □ | □ | □ | □ | □ | □ |
| 12. Children with EBD would hurt themselves or other children. | □ | □ | □ | □ | □ | □ |
| 13. When children have problems with their emotions and behavior, it is because their parents did not raise them properly. | □ | □ | □ | □ | □ | □ |
| 14. Children with EBD are troublemakers. | □ | □ | □ | □ | □ | □ |
